# A Novel Spatial Feature for the Identification of Motor Tasks Using High-Density Electromyography

**DOI:** 10.3390/s17071597

**Published:** 2017-07-08

**Authors:** Mislav Jordanić, Mónica Rojas-Martínez, Miguel Angel Mañanas, Joan Francesc Alonso, Hamid Reza Marateb

**Affiliations:** 1 Department of Automatic Control (ESAII), Biomedical Engineering Research Centre (CREB), Universitat Politècnica de Catalunya (UPC), Barcelona 08028, Spain; mislav.jordanic@upc.edu (M.J.); mmrojasm@unbosque.edu.co (M.R.-M.); joan.francesc.alonso@upc.edu (J.F.A.); h.marateb@eng.ui.ac.ir (H.R.M.); 2 Biomedical Research Networking Center in Bioengineering, Biomaterials and Nanomedicine (CIBER-BBN), Madrid 28029, Spain; 3 Bioengineering Department, El Bosque University, Bogotá 110121, Colombia; 4 Biomedical Engineering Department, Engineering Faculty, University of Isfahan, Isfahan 81746-73441, Iran

**Keywords:** high-density electromyography, pattern recognition, myoelectric control, mean shift, prosthetics

## Abstract

Estimation of neuromuscular intention using electromyography (EMG) and pattern recognition is still an open problem. One of the reasons is that the pattern-recognition approach is greatly influenced by temporal changes in electromyograms caused by the variations in the conductivity of the skin and/or electrodes, or physiological changes such as muscle fatigue. This paper proposes novel features for task identification extracted from the high-density electromyographic signal (HD-EMG) by applying the mean shift channel selection algorithm evaluated using a simple and fast classifier-linear discriminant analysis. HD-EMG was recorded from eight subjects during four upper-limb isometric motor tasks (flexion/extension, supination/pronation of the forearm) at three different levels of effort. Task and effort level identification showed very high classification rates in all cases. This new feature performed remarkably well particularly in the identification at very low effort levels. This could be a step towards the natural control in everyday applications where a subject could use low levels of effort to achieve motor tasks. Furthermore, it ensures reliable identification even in the presence of myoelectric fatigue and showed robustness to temporal changes in EMG, which could make it suitable in long-term applications.

## 1. Introduction

Electromyography (EMG) is a technique for recording the electrical activity produced by skeletal muscles. The EMG signal is a summation of action potentials produced by muscle fibers, directly triggered by the action potentials traveling along motor neurons [1]. Since EMG is an important source of neural information, it has been extensively studied in the field of human-machine interfacing [2,3]. Applications of EMG include the control of neurorehabilitation devices such as prostheses [4,5], rehabilitation robots [6,7], and identification of muscle anatomical structure [8], but also implementations in leisure activities such as sports [9] and computer games [10].

EMG signals could be recorded either non-invasively (surface EMG, sEMG) or invasively with needle and wire electrodes (intramuscular EMG, iEMG) [11]. Although the iEMG has higher signal-to-noise ratio, both approaches provide a similar quality of identification of upper-arm motor task [12]. Moreover, sEMG is preferred as it is recorded non-invasively.

The pattern recognition approach has been recently used in research laboratories as a state-of-the-art method to decode neural information. Its main advantage over conventional systems is the instant activation of a task belonging to any of the available degrees-of-freedom (DoFs). Many classifiers such as linear discriminant analysis (LDA), support vector machine, and artificial neural network were successfully employed for this purpose with a high identification fidelity [13], but many authors agree that the choice of the features is more important than the choice of the classifier [12]. Hence, simple and fast classifiers are preferred, among which the LDA is commonly used and has become a general recommendation [3,14]. In addition, different studies have focused on pattern recognition from the analysis of isometric contractions for myoelectric control, especially when considering subjects with neuromuscular impairment (in stroke for example) [15] and even for prostheses control for amputees [16]. Additional examples can be found in [17,18,19].

Features can be calculated in time, frequency/scale, and time-frequency/scale domain [2,3,13]. Time domain features are usually used because of their computational simplicity and good performance [3]. Additionally, they can be combined with other features to increase the performance, e.g., autoregressive features [12].

The influence of the physiological (e.g., muscle fatigue) or non-physiological (electrode-skin impedance) non-stationarity of the EMG features is a big issue in neuromuscular control. As a solution, Vidovic et al. [20] and Hahne et al. [21] proposed a real-time retraining of the classifier where the parameters are constantly updated. Liu et al. [22] proposed a universal LDA classifier which was trained during different days and then combined. Such methods adapt the model to changes in the features, rather than using robust features.

Moreover, the variation of force can affect the identification [23]. Scheme and Englehart [24] recommended to train the classifier using all effort levels, whereas He et al. [25] tackled the problem using a feature set based on the frequency content of the signal and muscle coordination.

With the recent introduction of high-density EMG (HD-EMG) [26], i.e., multichannel EMG recorded using 2D grids of closely spaced sEMG electrodes, multiple studies have reported improvement in task identification. Stango et al. reported that spatial features extracted from the HD-EMG are robust to the electrodes shift. Geng et al. [27] and Du et al. [28] exploited the power of deep convolutional network to design gesture recognition classifier that classifies instantaneous maps, i.e., raw HD-EMG samples. Hahne et al. extracted features using spatial filters optimized to increase separability between different classes [29]. This methods exploit the information about spatial muscle activation pattern extracted from the HD-EMG and the fact that the myoelectric activity over different parts of muscle depends on the various factors (e.g., contraction level [30], duration of the contraction [31], and joint position [32]) and can be useful in differentiation between tasks.

In our previous work, we used the center of gravity as a feature to describe spatial patterns in HD-EMG [18,19,33]. In this work, we propose a new spatial feature for task identification based on the modified mean shift algorithm. Novel features were evaluated in the identification of four isometric motor tasks of the upper-limb (flexion/extension, supination/pronation of the forearm) using the LDA classifier. The proposed features were tested in three conditions: when training set and test set were recorded at the same time (time-dependent changes in the signal are minor), when test set was recorded after training set, and during the fatiguing exercise. In addition, features were tested during the identification of task recorded at different effort levels. The proposed features proved to improve the identification and are especially useful in extreme cases like identification of tasks recorded at very low effort level or identification of tasks during fatigue. These results confirm the usefulness of information of spatial distribution of myoelectric intensity over the muscle in discrimination between tasks.

The rest of the paper is organized as follows: in the next section, information about the experimental protocol and the task identification method used in this study is presented. Section 3 provides the results of the identification using the proposed features and its comparison with the previously established features. The discussion is provided in Section 4 and finally, the conclusions are summarized in Section 5.

## 2. Materials and Methods

### 2.1. Instrumentation and Measurement Protocol

Eight healthy subjects (age: 36 ± 5 years; height: 177 ± 5 cm; weight: 75 ± 9 kg; body mass index: 23.7 ± 2.3) participated in the experiment. They reported no pain, and previously had not suffered any injuries or neuromuscular upper limb impairments. The study was conducted in accordance with the Declaration of Helsinki and subsequent amendments concerning research in humans and was approved by the University Ethics Committee and the local government. Recordings and results were documented with the registration number, which corresponded to the Spanish ministry project MICINN (TEC2008-02274): “Analysis of the dynamic interactions in non-invasive multichannel biosignals for rehabilitation and therapy”. All subjects gave their written informed consent to participate in the experimental protocol.

**Figure 1:**
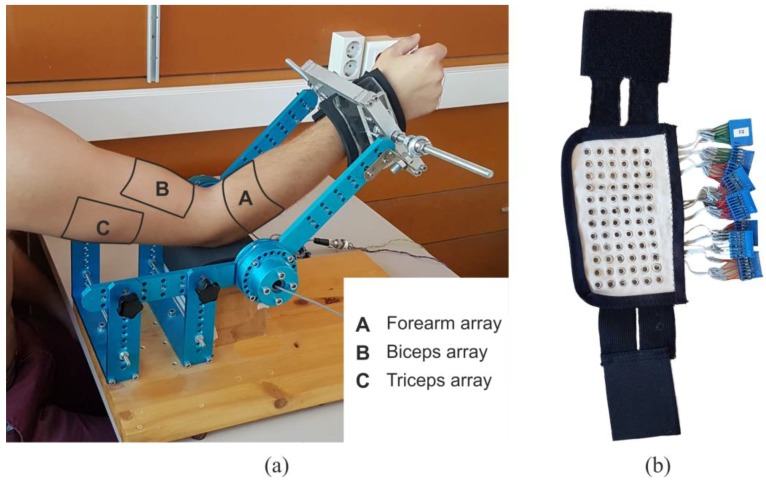
Figure shows (**a**) the position of the arm in the mechanical brace during the recording with the marked outlines of the electrode arrays; and (**b**) an electrode array.

Subjects performed four different isometric upper-limb tasks with two degrees of freedom: flexion/extension and supination/pronation of the forearm. During the experiment they were seated upright with their back being straight. Their dominant arm was positioned in the sagittal plane with the elbow flexed at 45 degrees and the forearm positioned in the middle between supination and pronation, thumb pointing upwards (Figure 1). To avoid muscle activation due to gripping, their hands were fixed at the wrist using a mechanical brace. The brace also contained two torque meters that measured exerted torque at the elbow joint.

HD-EMG was measured on five superficial muscles involved in the presented tasks: biceps brachii, triceps brachii, brachioradialis, anconeus, and pronator teres. Signals were recorded using three two-dimensional electrode arrays manufactured as silver-plated eyelets (2.5 mm radius) positioned in a quadrature grid with a 10 mm inter-electrode distance and embedded in a non-conductive fabric (Figure 1a).

After the skin was shaved, cleaned, and treated with abrasive gel, the following electrode arrays were positioned over the upper limb using elastic straps: two electrode arrays (dimensions: 8 rows × 15 columns) were positioned on the upper arm covering biceps brachii and triceps brachii muscles. The center of each electrode array was placed according to the positions recommended by the SENIAM project [34]. The third electrode array was placed over the forearm, with the first row of electrodes approximately 2 cm below elbow crest, covering brachioradialis, anconeus, and pronator teres muscles. A line connecting the origin and insertion of the targeted muscles were previously marked on the skin and the electrode array was placed to optimally cover these muscles. The forearm electrode array had six rows and between 17 and 19 columns, depending on the forearm circumference. After positioning the electrodes, the conductive gel was applied through the eyelet of each electrode (20 µL) using the dosimeter (Multipette Plus, Eppendorf, Germany).

HD-EMG signals were recorded in monopolar mode using three commercially available amplifiers with simultaneous sampling (EMG-USB, 128 channels, 2048 Hz sampling frequency, 10–750 Hz passband, manufacturer LISiN-OT Bioelettronica, Turin, Italy). Torque exerted on the elbow joint was measured using two torque transducers (OT Bioelettronica, range 150 Nm) and was displayed to the patient in real time. The detailed information on the instrumentation settings can be found in [35].

Prior to the experiment, the maximal voluntary contraction (MVC) was measured for each task as the maximal of three consecutive trials. In the first part of the experiment subjects were instructed to perform four defined tasks at three randomized different effort levels: 10% MVC, 30% MVC, and 50% MVC. Having been instructed to maintain the target level for 10 s, the exerted torque was displayed to the subjects. Tasks were performed in random order and between two consecutive recordings there was a two-minute rest to prevent cumulative fatigue.

Approximately 30 min (33 ± 3 min) after the first part of the protocol, endurance measurements were performed. Subjects were instructed to perform each task at 50% MVC until failure. After each measurement, subjects rested for five min.

### 2.2. HD-EMG Processing

The recorded HD-EMG signals were band-pass filtered using a 4th order Butterworth filter, with the cut-off frequencies of 15 Hz and 350 Hz, in the forward and reverse direction as to minimize the distortions. Outlier channels were automatically identified using a previously described algorithm [35].

HD-EMG recordings were divided into non-overlapping 150 ms time windows and the average HD-EMG activation maps were then calculated for each window in all three electrode arrays (biceps, triceps, forearm) using the RMS values. Activation maps can be conceptually perceived as images where pixels correspond to channels, and pixel intensities correspond to the muscle activation map in each channel. They were calculated as:

(1) $$AM_{i,j}=\sqrt{\frac{1}{N}\overset{N-1}{\underset{n=0}{\sum }}EMG_{i,j}^{2}\left[n\right]}$$

where *AM* is the activation map, *N* corresponds to the number of samples in each window (given a sampling frequency of 2048 Hz, *N* = 410), and *EMG_i,j_* denotes the EMG signal recorded by the electrode located at (*i,j*) position in the recording array. Pixels in AM corresponding to the outlier channels previously identified as artifacts were discarded and substituted using the triangular interpolation [35]. Examples of torque and EMG signals can be found in the Appendix B.

### 2.3. Feature Extraction

**Figure 2:**
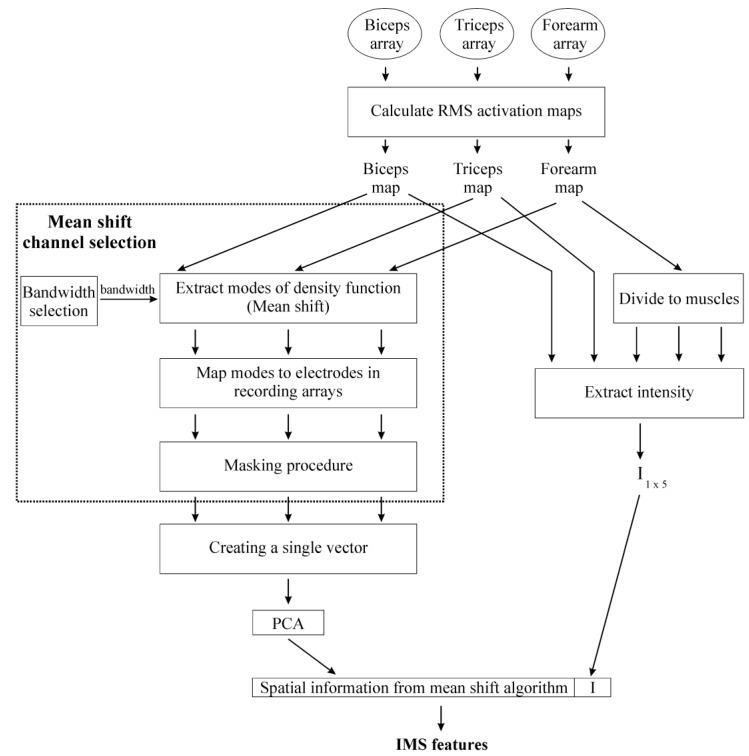
Feature extraction flowchart.

Identification was performed using the combination of intensity features and spatial features (Figure 2). Spatial features were extracted using the mean shift algorithm [36], a non-parametric approach to estimate modes (local maxima) of the underlying density function by an iterative procedure. The details of the mean shift algorithm are provided in the Appendix A and are briefly discussed here. A centroid point *y* was positioned at a random point in the space and the mean value was calculated for all points *x*, which were located within the Euclidean distance, i.e., bandwidth *h*, from the current centroid. This mean value was assigned as a new position of a centroid *y* in the next iteration. The procedure can be mathematically defined as:

(2) $$y_{i+1}:={\frac{\sum_{j=1}^{M}x_{j}}{M}|}_{\forall x\;s.t.\;\;\|x-y_{i}\|\leq h}$$

where *x_j_* (*j* = 1, 2, …, *M*) are samples of the unknown distribution, *y_i_* is the centroid in the ith iteration of the algorithm and the *h* is a bandwidth parameter. The algorithm stops when the position of the centroid (*y*) remains constant in consecutive iterations (up to a tolerance). This centroid *y* is considered to be a mode of the underlying density function. In this study, modes of the density function of RMS activation maps were found using the mean shift algorithm implemented in Python [37] and were used as features in the identification.

The bandwidth *h* was estimated automatically for each map. The maximum Euclidean distance between *k* nearest neighbors (where k was set to 50% of the total number of elements in the map) was calculated for every sample and the average of the maximum distances was calculated. The bandwidth used in this paper was obtained by multiplying this average distance by a bandwidth factor of 0.5, selected as a tradeoff between the amount of information and the processing time.

Prior to using the mean shift algorithm, each RMS activation map was transformed to a matrix of n rows, each row a channel, by three columns where the first two corresponded to the *x*, and *y* location of the channel in the activation map and the third to its intensity as estimated from the RMS of the signal. Since we used the spherical kernel, i.e., the bandwidth *h* had an equal value in all three dimensions, data was standardized to have zero mean and unity variance in all three dimensions.

A matrix of zeros with the same dimension of the electrode array was then created. Each mode detected by the mean shift algorithm was mapped to the closest location of the electrode in the array and its value was set to one. The result of this step was a binary image where the number of nonzero elements was equal to the number of detected modes. The procedure was repeated for all three activation maps. The resulting matrices were reshaped as a single 1-d vector in which the number of elements equaled to the total number of recorded EMG channels (for all three electrode arrays).

Principal component analysis (PCA) was then used for reducing the dimensionality of the feature space. A cumulative percentage of variance of 90% was used for dimensionality reduction, i.e., after the transformation to the orthonormal space, features were ordered by variance, and only the features explaining at least 90% of the cumulative variance were kept [38]. This reduced spatial feature set was then combined with the intensity features.

For calculation of intensity features, HD-EMG activation maps were segmented into areas covering the targeted muscle following the same procedure described in [35] and repeated in [33]. Segmentation discards the map areas not covering the recorded muscle (e.g., edges of maps), and also divides the forearm map into three different maps which correspond to forearm muscles. From the resulting five segmented activation maps (biceps brachii, triceps brachii, brachioradialis, anconeus, and pronator teres), intensity features (I) were calculated as:

(3) $$I=log_{10}\frac{1}{N}\sum_{i,j}SAM_{i,j}$$

where *I* is the intensity feature, *SAM_i,j_* is the intensity value of the pixel at location (*i,j*) in the segmented activation map *SAM*, and *N* is the total number of pixels in that map. Therefore, this procedure extracts five intensity features, one for each muscle. By concatenation, these intensity features were combined with the reduced spatial features into a single feature vector. These generated features were used in the identification and will be referred to as IMS from now on.

Results were compared with the previously proposed feature set: a combination of intensity and center of gravity (ICG) of segmented activation maps [18,19,33]. In this feature set, the center of gravity represents the traditional approach of describing the spatial information of intensity distribution over the muscle. The center of gravity (CG) has two dimensions and was calculated for each of the five muscles as:

(4) $$CG=\;\frac{\sum_{i,j}SAM_{i,j}\left[\begin{array}{c} i\\ j \end{array}\right]}{\sum_{i,j}SAM_{i,j}}$$

Therefore, ICG is a feature vector of 15 dimensions. Identification was also performed using only intensity features (I), and two classical features, single differential signal (Diff) and time-domain features (TD). One differential signal was obtained from each of five muscles using a pair of electrodes selected within the electrode arrays. Two adjacent electrodes located over the location proposed by the SENIAM were used to obtain the differential signal. Feature used in the analysis is RMS value of the differential signal calculated over the 150 ms time window. On the other hand, five TD features were calculated for each recorded channel. These features were firstly proposed by Hudgins [4] and used many times in literature (for example [39]). They were: RMS value, mean absolute value, number of zero crossings, waveform length, and number of slope sign changes. To be reduced in number, obtained features were projected to the space of lower dimensionality using PCA. As for the calculation of MS, only projections explaining 90% of variance were kept.

### 2.4. Task Identification

LDA was used for the identification of motor tasks. Task identification was evaluated using the repeated holdout method (N = 20). Observations were randomly assigned to the training set and the test set (70% to the training set) using stratified sampling. Both the PCA transformation function and the LDA discriminant function were calculated on the training set, and evaluated on the test set. Only the results of the test set are presented. Identification results were expressed in terms of sensitivity (S) and precision (P), defined as:

(5) $$S=\;\frac{TP}{TP+FN}$$

(6) $$P=\;\frac{TP}{TP+FP}$$

where TP (true positive) is the number of samples that were correctly classified, FN (false negative) is the number of samples belonging to a certain class and erroneously classified into another class, whereas FP (false positive) is the number of samples incorrectly classified to a certain class [39].

The identification was evaluated under various conditions:

- Short-term identification
- Long term identification
- Identification during fatigue

In short term identification, the training and validation sets were recorded at the same time. This are in fact the “perfect conditions” where the slow time-dependent changes in the sEMG signal associated with the recordings were minor. The dataset was composed of the recordings obtained in the first part of the measurement protocol.

Two types of identification were tested: identification of task and identification of task and effort level. In the identification of task, only the task was identified, regardless of the effort level, i.e., recordings of different effort levels were pooled together to form a single class. In this experiment, there were only four classes: flexion, extension, supination, and pronation. Identification of task and effort level was designed as a two-step classifier. In the first step the task was identified, regardless of the effort level, as discussed above. In the second step, classification of three levels of effort was performed for each identified task individually. The second step consisted of four different classifiers, one classifier for the identification of the effort level of each task. For identification of effort level of a sample, the second step classifier was selected depending on the classified task in the first step [19]. Classifiers in the second step were designed using the reduced feature set, as proposed in [19], where features were extracted from agonist-antagonist muscle pairs involved in the selected task, i.e., biceps brachii and triceps brachii for identification of the effort level during flexion and extension; biceps brachii, brachioradialis and anconeus for supination; and pronator teres and anconeus for pronation. Since the modes of the density function were calculated for the entire forearm array (not for each muscle separately), modes extracted from the entire forearm array were used in the identification of the effort level during supination and pronation.

In the long-term identification, robustness to time effect was tested. In this part of the protocol, the training set was composed of all observations recorded in the first part of the measurement protocol, whereas the test set was composed of the first two seconds of the recordings in the second part of the protocol. Having in mind that there was a time gap between the first part of the protocol and the second part of the protocol (≈30 min), using this procedure the influence of different time effects can be evaluated (e.g., drying of conductive gel). On the other hand, to prevent the effect of fatigue, only the first two seconds of the total duration of the exercise were used in the test set.

Robustness of the identification was also tested during endurance tasks recorded during the second part of the recording protocol. Recordings were divided into five equal time epochs. The classifier was trained using the samples extracted from the first 20% of the total duration of recording (TDR), and was evaluated on five equally long segments throughout the exercise: 0–20% TDR, 20–40% TDR, 40–60% TDR, 60–80% TDR, and 80–100% TDR.

### 2.5. Statistical Methods

Statistical difference in performance was checked between IMS and other feature sets. The Kolmogorov-Smirnov test showed that the data significantly deviate from a normal distribution, so the non-parametric statistical Wilcoxon signed rank test was used to test for differences between distributions. In addition, the non-parametric repeated measures Friedman test was used to test the differences in identification of the task when the training set was composed of pool of all effort levels, and test set of only 10% MVC, 30% MVC, or 50% MVC. This was repeated for all feature sets. The significance level was set to *p* = 0.05. Statistical tests were performed using the IBM SPSS Statistics software package (IBM SPSS Statistics for Windows, version 20.0, released 2011; IBM Corp.: Armonk, NY, USA).

## 3. Results

### 3.1. Bandwidth and Time Window Selection

**Figure 3:**
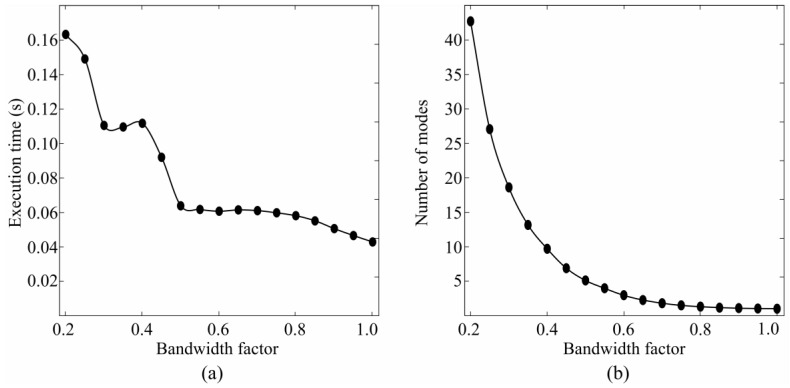
Figure shows average processing time (**a**) and number of estimated modes (**b**) of mean shift algorithm given the specific bandwidth factor in the range from 0.2 to 1.

Two aspects where considered in the choice of the bandwidth factor: the average execution time of the mean shift algorithm and the amount of information, i.e., number of detected modes (Figure 3). The average processing time was measured on a standard desktop computer featuring an Intel^®^ E8400 Core^TM^ 2 Duo CPU (Intel, Santa Clara, CA, USA). Both graphs show that the elbow point was at the bandwidth factor of 0.5. If the bandwidth factor is set to a lower value, both the execution time and the number of modes increase notably. A rapid increase of the number of modes for lower bandwidths implies that the mean shift algorithm is focused on local maxima, whereas the increase of the execution time increases the latency of the system. On the other hand, there was not much difference when the bandwidth factor ranges between 0.5 and 1.0, both in the number of estimated modes, and the execution time. Therefore, the range from 0.5 to 1.0 was considered of interest for the selection of the bandwidth factor.

**Figure 4:**
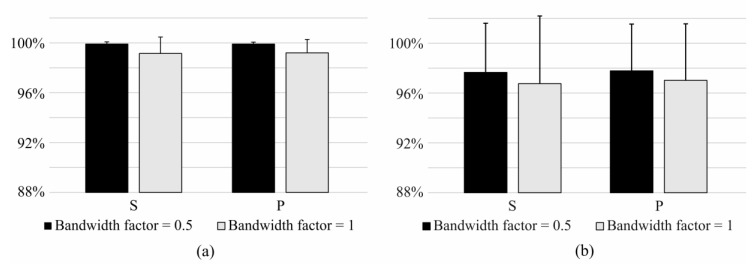
Sensitivity and precision of short-term identification of (**a**) identification of task and (**b**) identification of task and effort level using bandwidth factors 0.5 and 1.0 in mean shift algorithm.

The identification of task and the identification of task and effort level (Figure 4) were compared using the bandwidth factor of 0.5 and 1.0. The performance of the algorithm was significantly higher when using the bandwidth of 0.5 compared with that of 1 (*p* < 0.05).

**Figure 5:**
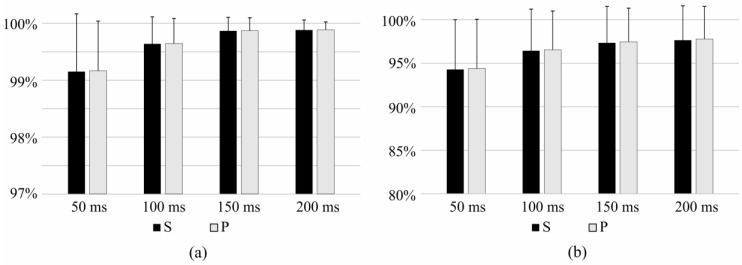
Sensitivity (S) and precision (P) of (**a**) identification of task and (**b**) identification of task and effort level for time windows of 50 ms, 100 ms, 150 ms, and 200 ms.

On the other hand, the effect of duration of time window in which the features were calculated was analyzed and results are presented in Figure 5. Identification based on the IMS features extracted from the 150 ms and 200 ms time windows significantly outperform the identification when features were extracted from shorter time windows, whereas no significant difference was found between results calculated on 150 ms and 200 ms windows.

Consequently, the bandwidth factor of 0.5 and the time widnow of 150 ms were used in the rest of the paper.

### 3.2. Short-Term Identification

**Figure 6:**
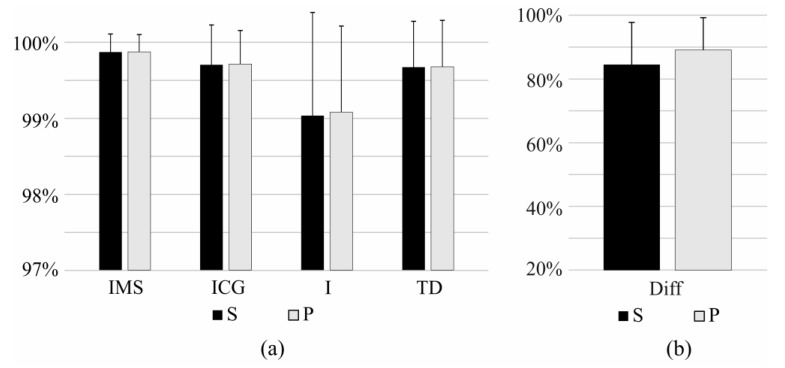
Sensitivity (S) and precision (P) of short-term identification of task using (**a**) IMS, ICG, I, and TD features, and (**b**) using Diff features. Results of the identification using Diff is showed in a different scale.

Table 1 shows the results of the identification of task using the novel features proposed in this paper and Figure 6 shows the comparison between IMS, ICG, I, TD, an Diff features in the identification of tasks. IMS significantly outperformed all of the compared features (*p* < 0.05).

**Figure 7:**
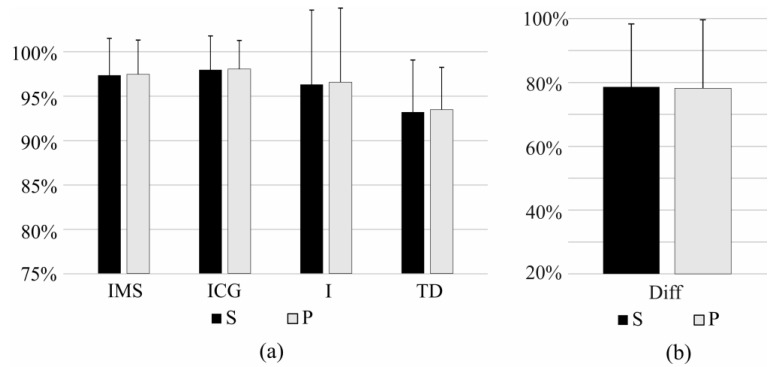
Sensitivity (S) and precision (P) of short-term identification of task and effort level using (**a**) IMS, ICG, I, and TD features, and (**b**) using Diff features. Results of the identification using Diff is showed in a different scale.

The results of the identification of the task and effort level using IMS features are given in Table 2, whereas comparison between IMS and other features is shown in Figure 7. IMS features significantly outperform I, TD, and Diff features (*p* < 0.05), whereas the ICG features slightly outperform IMS features (ΔS = 0.6%, ΔP = 0.6%; *p* < 0.05).

**Figure 8:**
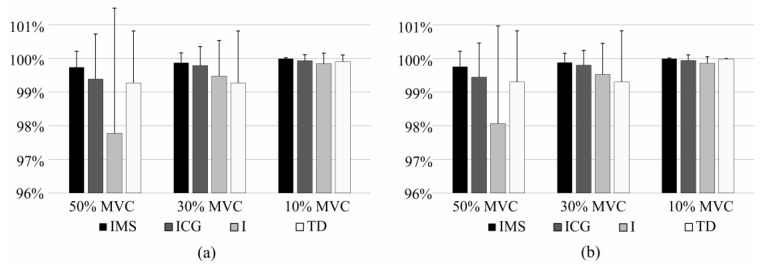
Figure shows sensitivity (**a**) and precision (**b**) of short-term identification of task recorded at specific effort level using IMS, ICG, I, and TD features.

**Figure 9:**
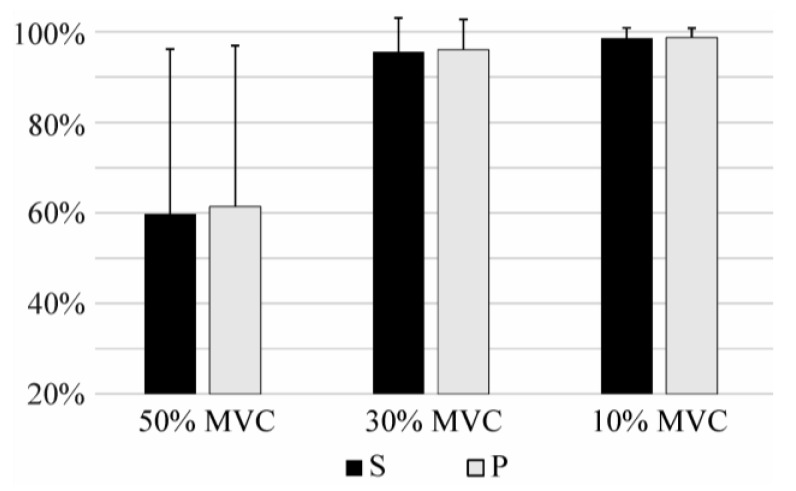
Figure shows sensitivity (S) and precision (P) of short-term identification of task recorded at specific effort level using Diff features.

The sensitivity and precision of the task identification when the classifier was trained using all effort levels (pool of 10%, 30%, 50% MVC) and tested using a specific effort level can be seen in Figure 8 and Figure 9 for comparison of IMS, ICG, I, and TD features, and for Diff features, respectively. This experiment shows how well each feature set identifies the task of a specific effort level. The difference in performance is especially pronounced in the identification of tasks at very low effort level (10% MVC). IMS significantly outperforms I and Diff features at all effort levels, but the difference between IMS and ICG features and the difference between IMS and TD are not significant at moderate effort levels (30% MVC and 50% MVC), whereas IMS features are specifically and significantly better when identifying tasks at low effort levels (10% MVC).

Additionally, no significant difference between task identification at three different effort levels was seen when using IMS features, whereas these differences were significant for other feature sets. This could mean that these novel IMS features are more robust to the variation in the effort level.

### 3.3. Long-Term Identification

**Figure 10:**
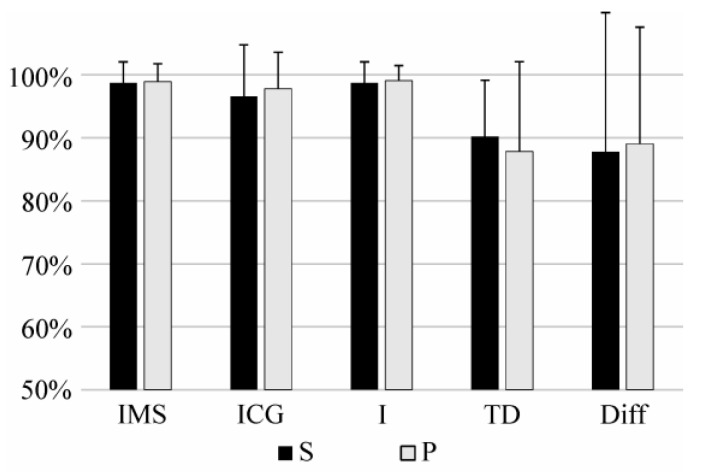
Sensitivity (S) and precision (P) of long-term identification of task using IMS, ICG, I, TD, and Diff features.

Identification was tested when a significant amount of time passed between the recording of the training and test sets. This allowed an evaluation of influence of slow time-dependent changes in the EMG signal on the robustness of the identification. Figure 10 shows the comparison of the intensity features and the combination of intensity and spatial features when these last ones were calculated as the center of gravity or using the mean shift algorithm. There are no significant differences in performances between these IMS, ICG, and I features, whereas IMS feature significantly outperform TD and Diff features (*p* < 0.05). However, it should be noted that the test set was composed only of samples recorded at 50% MVC. And, as previously proven in literature [18], and shown in Figure 8 and Figure 9, the use of spatial information is particularly useful in contractions at low effort levels, whereas only intensity can be sufficient to successfully identify contractions of moderate effort levels (as 50% MVC).

### 3.4. Identification During Fatigue

The influence of fatigue on EMG was evaluated using endurance recordings. Recordings were divided into five equal time epochs. The training set was obtained from the first epoch (0–20% TDR), and the identification was performed on all five time epochs.

**Figure 11:**
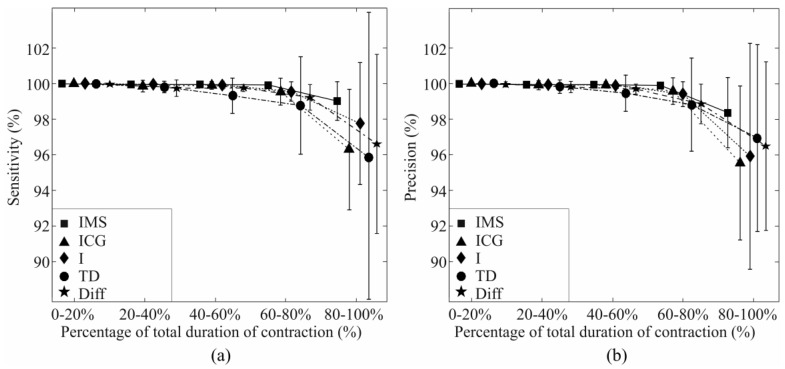
Influence of fatigue on (**a**) sensitivity and (**b**) precision of the identification of task using IMS, ICG, I, TD, and Diff features.

Changes of sensitivity and precision during the exercise can be seen in Figure 11. It can be seen how all feature sets perform similarly at the beginning of the contraction, whereas identification indices decay towards the end as the fatigue accumulates. However at the final stages of fatigue (80%–100% TDR) IMS features significantly outperform other feature sets (*p* < 0.05). These results show the robustness of the IMS features to the fatigue.

## 4. Discussion

This study showed that the combination of intensity and spatial information is useful for the extraction of neuromuscular information. The spatial information was calculated from the RMS activation maps using the mean shift algorithm. Results were evaluated using the 70% repeated holdout method and stratified sampling as to have sufficient number of samples of each class in the sets. To prevent the type III statistical error [40,41], a repeated hold-out was used. Sensitivity and precision, as appropriate unbiased measures in analyzing imbalanced multi-class problems [19,33], were used to quantify the identification.

IMS features achieved very good results compared to other feature sets during task identification when the task was performed at very low effort level. Moreover, the Friedman test showed no significant differences in task identification using IMS when tasks were performed at 10% MVC, 30% MVC, or 50% MVC. This can be a very important quality in everyday applications where subject could not need to contract muscles at moderate effort level to complete the task. It can be a step toward more natural control where even slight contractions can be successfully identified. In fact, only activations with low level of intensity are sometimes possible in patients with neuromuscular impairments.

A high identification rate is not the only factor important in the extraction of neural information from sEMG. The system should also be robust to slow time-dependent changes such as fatigue and electrode-skin contact impedance [42]. Therefore, the robustness of the proposed features was tested with respect to time and fatigue. When evaluating the time effect, no significant differences in performance were found between IMS, ICG, and I feature sets and IMS significantly outperformed TD and Diff features. However, time effect was evaluated only when test set was composed of contractions recorded at 50% MVC and, as shown in Figure 6, all features perform similarly for the identification of that effort level. This phenomenon was already remarked and described in [18] where authors noted that adding spatial features to intensity features significantly improved the identification of tasks recorded at low effort levels, whereas improvement is not significant at moderate effort levels. On the other hand, the proposed features are particularly robust in task identification during fatiguing exercises and show significantly higher identification rate when compared to other features. Further improvements in reliability of the identification during the long-term contractions and fatiguing contractions can be achieved by using adaptive identification models that are being constantly updated during the usage (e.g., [20,21,43]).

In the current work, features were extracted from the RMS activation maps of the HD-EMG. Although these features proved to be very effective, by describing the EMG signal with its RMS value, i.e., the estimator of variance, the information is partially lost. Since the gradient of the probability density function of raw EMG is a useful feature in task identification, statistical measures (e.g., modes) of the raw HD-EMG, i.e., joint distribution of instantaneous EMG amplitude over the electrode array, could provide valuable information. Moreover, in literature, features were often calculated for each channel separately and selected using the simple sequential method prior to classification [44,45]. On the other hand, Geng et al. recently proposed a more advanced channel selection method based on common spatial patterns [46]. Modes of the HD-EMG density function could be correlated with the channels with discriminative information and could be a useful tool in channel selection.

Finally, the mean shift algorithm can be used for clustering and, since it was shown that the algorithm is most effective in low-dimensional data, image segmentation is one of its most successful applications [36]. A mode of the density estimate, or in this case, a channel selected by the mean shift algorithm, can be considered as a cluster representative [47], related to the possible image segments, where spatial (pixel locations) and range features (the intensity of the grayscale value) are considered. The advantage of the mean shift is that it can be used for clustering non-convex shapes, albeit, it could segment complex non-convex regions in the activation maps. Since segmentation of the muscle activation map can improve the neuromuscular activity estimation [32], this could be a reason why mean shift features improved the performance of the movement detection system compared with previously published attributes. In addition, the algorithm only requires setting one parameter, bandwidth (*h*) and, unlike in the similar methods, it is not necessary to define the number of expected clusters. This is a big advantage because it does not require a priori knowledge on the number of clusters.

As a limitation of the study, it should be noted that the proposed features were tested only in highly controlled conditions of isometric contractions. The experiments during non-isometric contractions should be performed in order to validate the quality of the features in dynamic and more natural movements. Also, the experiment included only four tasks related to the elbow joint. Further analysis should include higher number of more complex tasks related to hand and shoulder. Moreover, all results were obtained during offline analysis. To evaluate practical aspects of the features, the experiment should be repeated using online identification and considering multiple transitions between tasks.

## 5. Conclusions

In conclusion, a new set of features for the identification of isometric motor tasks of upper limb was proposed. It was based on the combination of intensity and the spatial distribution of intensity of HD-EMG. These new features were evaluated using the LDA classifier and the results showed they improve the identification of tasks. Moreover, robustness of the features was tested under the influence of slow time-dependent changes of the EMG. They proved to be particularly useful for task identification when muscles were fatigued. The proposed methods could be used for the design and monitoring of rehabilitation therapies intended for patients with neuromuscular impairment, as well as for the control of external devices like exoskeletons, and prostheses.

## Figures and Tables

**Table 1:** Sensitivity and precision of identification of task using IMS features averaged between patients. Identification Indices for each patient were calculated as an average of hold-out repetitions (N = 20) and presented in terms of mean and standard deviation.

Task	Sensitivity	Precision
Flexion	99.7 ± 0.5%	99.9 ± 0.2%
Extension	99.9 ± 0.1%	99.9 ± 0.1%
Supination	99.9 ± 0.2%	99.7 ± 0.5%
Pronation	99.9 ± 0.1%	99.9 ± 0.1%
Average	99.9 ± 0.2%	99.9 ± 0.2%

**Table 2:** Sensitivity and precision of identification of task and effort level averaged between patients. Identification indices for each patient were calculated as an average of hold-out repetitions (N = 20) and presented in terms of mean and standard deviation.

Task	Sensitivity (%)	Precision (%)
Flexion 10% MVC	98.2 ± 2.8%	99.9 ± 0.3%
Flexion 30% MVC	98.7 ± 1.1%	97.0 ± 3.1%
Flexion 50% MVC	97.7 ± 2.9%	98.6 ± 1.1%
Extension 10% MVC	99.7 ± 0.6%	99.6 ± 1.1%
Extension 30% MVC	97.4 ± 3.4%	97.5 ± 2.1%
Extension 50% MVC	97.7 ± 2.3%	98.2 ± 2.9%
Supination 10% MVC	99.7 ± 0.5%	99.9 ± 0.2%
Supination 30% MVC	95.2 ± 7.1%	96.0 ± 5.1%
Supination 50% MVC	96.6 ± 4.9%	95.4 ± 6.3%
Pronation 10% MVC	99.8 ± 0.2%	99.4 ± 1.1%
Pronation 30% MVC	93.8 ± 12.3%	93.9 ± 11.3%
Pronation 50% MVC	93.7 ± 11.9%	94.2 ± 11.9%
Average	97.4 ± 4.2%	97.5 ± 3.9%

**Figure A1:**
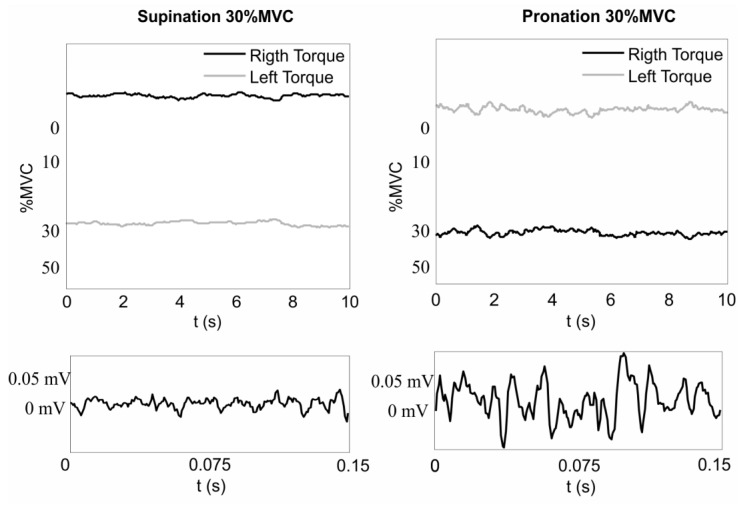
Example of torque and EMG signals in supination and pronation in one subject. Left. Supination at 30% MVC. The exerted torque on right (black) and left (gray) sides of the mechanical brace are shown at the top of the figure. The sEMG signal recorded on one of the channels of the Pronator Teres muscle is shown at the bottom. Rigth. Torque signals for Pronation at 30% MVC are shown on the top of the figure. The sEMG signal recorded on the same channel as in the previous case is shown at the bottom.

**Figure A2:**
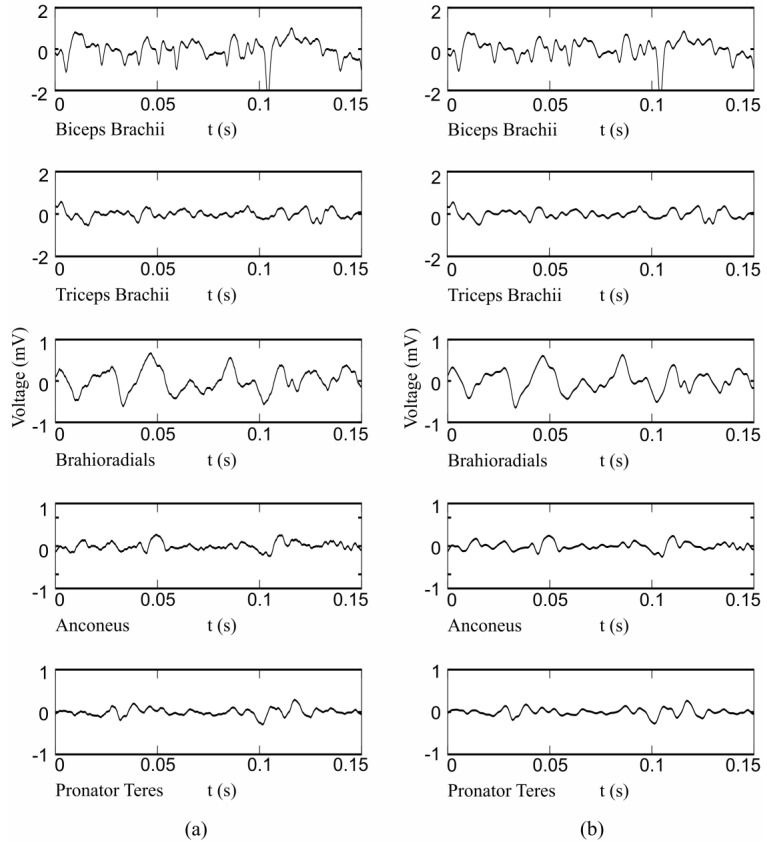
Examples of recorded EMG signals from five muscles (biceps brachii, triceps brachi, brachioradialis, anconeus, bracioradialis, and pronator teres) during flexion. Figure shows (**a**) raw signals and (**b**) signals filtered using 4th order Butterworth filter with the cut-off frequencies of 15 Hz and 350 Hz.

**Figure A3:**
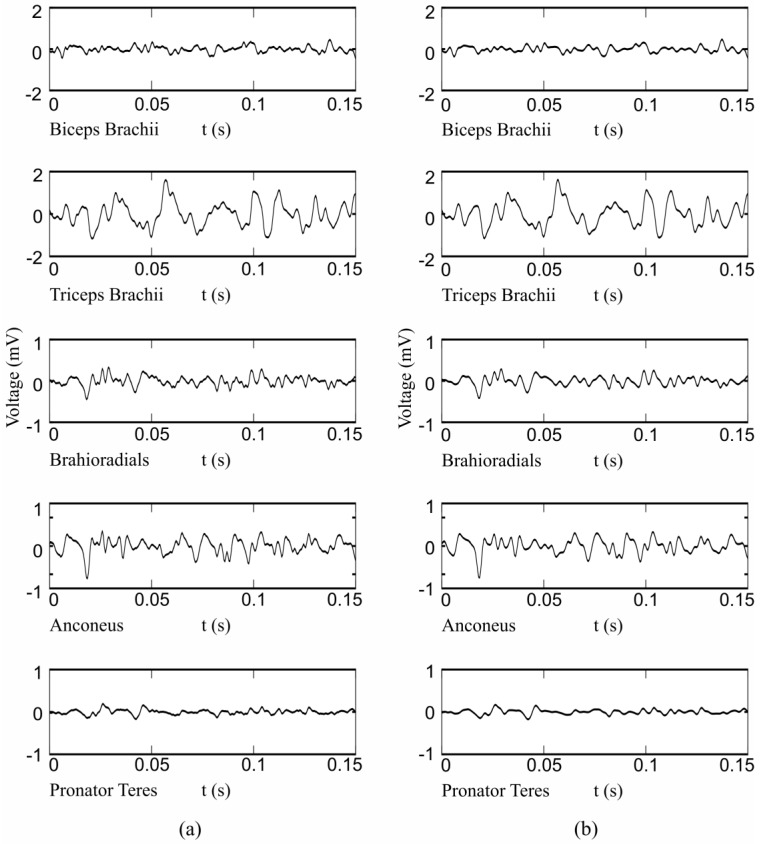
Example of recorded EMG signals from five muscles (biceps brachii, triceps brachi, brachioradialis, anconeus, bracioradialis, and pronator teres) during extension. Figure shows (**a**) raw signals and (**b**) signals filtered using 4th order Butterworth filter with the cut-off frequencies of 15 Hz and 350 Hz.

**Figure A4:**
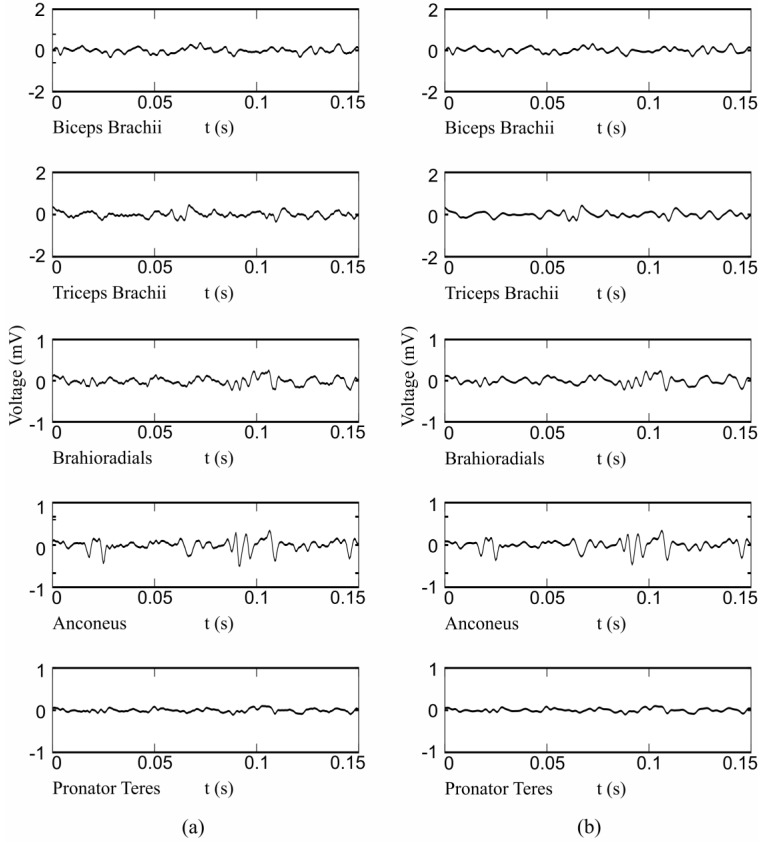
Example of recorded EMG signals from five muscles (biceps brachii, triceps brachi, brachioradialis, anconeus, bracioradialis, and pronator teres) during supination. Figure shows (**a**) raw signals and (**b**) signals filtered using 4th order Butterworth filter with the cut-off frequencies of 15 Hz and 350 Hz.

**Figure A5:**
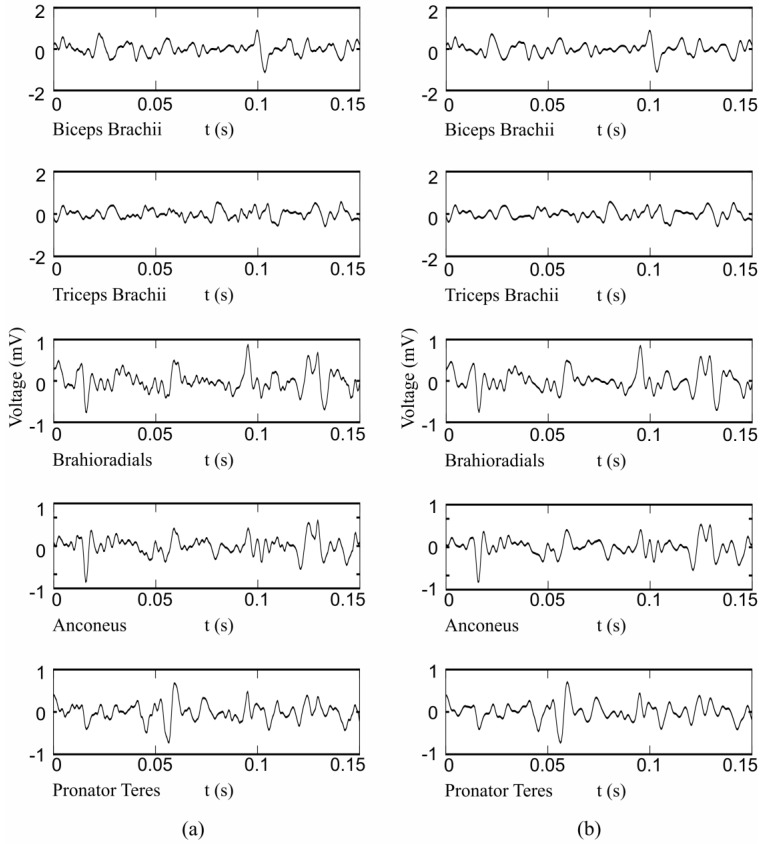
Example of recorded EMG signals from five muscles (biceps brachii, triceps brachi, brachioradialis, anconeus, bracioradialis, and pronator teres) during pronation. Figure shows (**a**) raw signals and (**b**) signals filtered using 4th order Butterworth filter with the cut-off frequencies of 15 Hz and 350 Hz.

## Appendix A

The mean shift algorithm is a non-parametric approach to estimate the gradient of a density function. It was first proposed by Fukunaga and Hostetler [48] in 1975, but did not get a lot of attention of the academic community initially. Although their work was cited more than 1500 times in literature, most of the cites occurred after the famous publication of Comaniciu and Meer [36] in 2002 (counting almost 6000 citations) that revised the method and drew attention of the scientific community to it.

The algorithm is the enhanced version of the Parzen window technique for the estimation of density using a kernel [49] and its extension to multivariate distributions [50], given that density for the point **x** can be estimated based on the observed samples **x***_i_* (*i* = 1,2,…,*n*) using the kernel function *K* as:

(A1) $$\hat{f}\left(x\right)\;=\;\frac{1}{n}\overset{n}{\underset{i=1}{\sum }}K_{H}\left(x-x_{i}\right)$$

(A2) KH=|H|−12 K(H−12 x)

where *f̂(***x***)* is the estimated density, *K_H_* is the normalized kernel function, and *H* is *d x d* bandwidth matrix. The bandwidth matrix *H* can be fully parameterized, diagonal, or, as in this paper, proportional to identity matrix (***H*** *=* *h**I***), which simplifies the expression for the density estimation to:

(A3) $$\hat{f}\left(x\right)\;=\;\frac{1}{nh^{d}}\overset{n}{\underset{i=1}{\sum }}K\left(\frac{x-x_{i}}{h}\right)$$

where *f̂(***x***)* is the estimated density, *h* is a single bandwidth parameter, *d* is the number of dimensions, and *K* is the kernel function. Two commonly used univariate kernel profiles are Epanechnikov (*k_E_*) and Gaussian (*k_N_*):

(A4) kN(x)=e−12x,   x≥0

(A5) $$k_{E}\left(x\right)=\{\begin{array}{c} 1-x\;\;\;\;\;0\leq x\leq 1\\ 0\;\;\;\;\;\;\;\;\;\;\;\;\;\;\;\;\;\;\;\;x>1 \end{array}$$

which yield multivariate radially symmetric kernel (*K_E_*) and normal kernel (*K_N_*) respectively:

(A6) KN(x)=12πd/2e−12‖x‖2

(A7) $$K_{E}\left(x\right)=\{\begin{array}{cc} \frac{1}{2}\frac{d+2}{c_{d}}\left(1-{\left\|x\right\|}^{2}\right) & \;\;\;\;\;\;\;\|x\|\leq 1\\ 0\;\;\;\;\;\;\;\;\;\;\;\;\;\;\;\;\;\;\;\;\;\;\;\;\;\;\;\;\;\;\;\;\;\;\;\;\;\;\;\;\; & \|x\|>1 \end{array}$$

where *d* is the number of dimension and *c_d_* is the constant that ensures the kernel integrates to one.

Mean shift vector is defined as [36]:

(A8) $$ms\left(x\right)=\frac{\sum_{i=1}^{n}x_{i}g\left({\left\|\frac{x-x_{i}}{h}\right\|}^{2}\right)}{\sum_{i=1}^{n}g\left({\left\|\frac{x-x_{i}}{h}\right\|}^{2}\right)}-x$$

where *g(x)* is the negative derivative of the original univariate kernel profile *k(x):*

(A9) $$g\left(x\right)=-\frac{dk\left(x\right)}{dx}$$

Mean shift is a function defined for every point in space. It is a vector of difference between the current position and the weighted mean of all points within its bandwidth *h*, whose weights are defined by the kernel profile *g(x)*. Therefore, the mean shift vector always points to the direction of maximum increase of the density and can be considered as a function proportional to the gradient of the density function:

(A10) $$ms_{g}\left(x\right)\;\propto \;\nabla {\hat{f}}_{k}\left(x\right)$$

In addition, mean shift can be effectively used to find modes (local maxima) of the underlying density function by an iterative procedure. Kernel is usually centered at a random point in space and the mean shift vector is calculated. In the next iteration, the kernel is centered at the location pointed by the mean shift vector. The procedure is mathematically defined as:

(A11) $$y_{i+1}:=\frac{\sum_{j=1}^{n}x_{j}g\left({\left\|\frac{y_{i}-x_{j}}{h}\right\|}^{2}\right)}{\sum_{j=1}^{n}g\left({\left\|\frac{y_{i}-x_{j}}{h}\right\|}^{2}\right)}$$

By repeating this procedure, at every step, the center of the kernel is shifted to the direction of maximum increase of the density function until the local maximum is reached. At this location, the difference between two consecutive points is zero (up to a tolerance). These final stationary points are considered to be modes of the probability density function:

(A12) $$y_{i+1}-y_{i}=0$$

(A13) $$ms_{g}\left(y_{i}\right)=\nabla {\hat{f}}_{k}\left(y_{i}\right)=0$$

This algorithm is very useful in image processing and feature space analysis with many applications, of which clustering is the most popular. It only requires setting one parameter, bandwidth (*h*). On the other hand, unlike the similar methods, e.g., *k-* means clustering, it is not necessary to define the number of expected clusters. This is a big advantage because it does not require *a priori* knowledge on the number of clusters. Detailed explanation of the mean shift algorithm can be found in the literature [36,48].

In this study, modes of the density function of root-mean-square (RMS) activation maps were found using the mean shift algorithm implemented in Python [37] and were used as features in the identification. The Epanechnikov kernel profile was employed to describe the density function, which yielded flat kernel profile *g(x)* in the calculation of the mean shift vector:

(A14) $$g\left(x\right)=\{\begin{array}{cc} 1, & \;\;\|x\|\leq h\\ 0, & \;\;\|x\|>h \end{array}$$

This choice of the kernel profile simplified the update of the mean shift centroid to:

(A15) $$y_{i+1}:={\frac{\sum_{j=1}^{N}x_{j}}{N}|}_{\forall x\;s.t.\;\;\|x-y_{i}\|\leq h}$$

In the other words, the new centroid was calculated as the mean value of *N* points located within the Euclidean distance h from the current centroid.

## Appendix B

Example of the torque signals during supination and pronation can be seen in the Figure A1, along with the EMG signal recorded on pronator teres. It is possible to observe that the polarity of the torque signals change depending on the direction of the movement. The mechanical brace is fixed at the wrist so that the exerted force during supination and pronation is monitored by left and right torque meters, respectively. In addition, as expected, the amplitude of the sEMG signal in the Pronator Teres is higher during pronation.

On the other hand, examples of EMG signals recorded on five muscles during 30% MVC flexion, extension, supination, and pronation can be seen in Figure A2, Figure A3, Figure A4 and Figure A5, respectively. Figures show raw EMG signals and signals filtered using 4th order Butterworth filter with cut-off frequencies of of 15 Hz and 350 Hz. Scale for each muscle is the same across different tasks to show difference in EMG amplitudes in dependence of task.
